# Motivation to Motivate: Pilot of motivational interview training in a tertiary university hospital

**DOI:** 10.1192/j.eurpsy.2024.394

**Published:** 2024-08-27

**Authors:** K. Corrigan, G. Crudden, A. M. Clarke, A. Doherty

**Affiliations:** ^1^ St. Vincent’s University Hospital; ^2^Holles Street, The National Maternity Hospital, Dublin; ^3^Naas General Hospital, Kildare; ^4^The Mater Misericordiae Hospital, Dublin, Ireland

## Abstract

**Introduction:**

Motivational interviewing (MI) is an evidence-based communication style that is effective in facilitating behaviour change and patient engagement. Originally developed in the field of addiction, MI can be applied to address a range of health behaviours including smoking cessation, medication adherence and diabetes and weight management. Given its demonstrated efficacy, training clinicians in medical and psychiatry specialties in MI has potential to enhance patient outcomes.

**Objectives:**

1. Enhance awareness and understanding of the basic concepts and methods of MI among NCHDs (Non-Consultant Hospital Doctors) and hospital staff.

1. Assess the perceived effectiveness of the training and help foster a culture of teaching and learning within the hospital.

**Methods:**

MI training was organised by the psychiatry department and delivered by a certified external trainer. The training was structured into 2 sessions, each lasting three hours, with a six-week gap between sessions. The training was integrated into the regular academic teaching for psychiatry trainees and a circular email invite was sent to the medical NCHD cohort and psychology department. Pre and post-training questionnaires were collected from participants with five-point Likert scales used to gather responses

**Results:**

There were 40 attendees across the two sessions. In total, 25 questionnaire responses were collected for the pre training (62.5%) and 30 responses were collected post-training (75%).

Prior to training 48% (12/25) indicated they were familiar with MI, 48% felt confident in using MI and 88% (22/25) felt it was applicable to their practice. Post-training, 73% (22/30) felt confident in using MI, 90% (27/40) felt MI was applicable to their practice and 100% indicated they would use MI in their practice. The perception of applicability (p=0.011) and likely utilisation of MI skills (p<0.001) significantly increased over the course of the training as measured by paired t-test (n=23). Ninety-seven percent of responders stated they would recommend the training and 57% (17/30) indicated that they would use MI on a weekly basis in the future.

**Conclusions:**

NCHDs and other staff welcomed this training and indicated the training was relevant to their practice. MI demonstrated a positive effect on staff perceptions of applicability and future utilisation of MI skills. Increasing clinician self-perceived efficacy through training events may help contribute to a culture of learning and teaching in hospital settings.

**Disclosure of Interest:**

None Declared

